# A Simplified Method of the Assessment of Magnetic Anisotropy of Commonly Used Sapphire Substrates in SQUID Magnetometers

**DOI:** 10.3390/ma15238532

**Published:** 2022-11-30

**Authors:** Katarzyna Gas, Maciej Sawicki

**Affiliations:** Institute of Physics, Polish Academy of Sciences, Aleja Lotnikow 32/46, PL-02668 Warsaw, Poland

**Keywords:** sapphire, semiconductor substrates, magnetic anisotropy of sapphire, SQUID magnetometry, magnetic characterization, nanomaterials, 2D magnetism, dilute magnetic semiconductors, thin magnetic layers

## Abstract

Solid-state wafers are indispensable components in material science as substrates for epitaxial homo- or heterostructures or carriers for two-dimensional materials. However, reliable determination of magnetic properties of nanomaterials in volume magnetometry is frequently affected by unexpectedly rich magnetism of these substrates, including significant magnetic anisotropy. Here, we describe a simplified experimental routine of magnetic anisotropy assessment, which we exemplify and validate for epi-ready sapphire wafers from various sources. Both the strength and the sign of magnetic anisotropy are obtained from carefully designed temperature-dependent measurements, which mitigate all known pitfalls of volume SQUID magnetometry and are substantially faster than traditional approaches. Our measurements indicate that in all the samples, two types of net paramagnetic contributions coexist with diamagnetism. The first one can be as strong as 10% of the base diamagnetism of sapphire [−3.7(1) × 10^−7^ emu/gOe], and when exceeds 2%, it exhibits pronounced magnetic anisotropy, with the easy axis oriented perpendicularly to the face of c-plane wafers. The other is much weaker, but exhibits a ferromagnetic-like appearance. These findings form an important message that nonstandard magnetism of common substrates can significantly influence the results of precise magnetometry of nanoscale materials and that its existence must be taken for granted by both industry and academia.

## 1. Introduction

High crystalline quality is one of the key properties of bulk crystals that are to be used as solid-state substrates for the growth of (thin) homo- or heteroepitaxial structures [1], as well as for two dimensional (2D) materials [2]. Some of these structures are expected to become game changers in spintronics or may exhibit interesting magnetic properties from the fundamental point of view [3]. Among a few other characterization methods, integral magnetometry based on commercial superconducting quantum interferometer device (SQUID) magnetometers is employed to establish the magnetic constitution of these structures. This is where the real challenge begins, since the magnetic response of these nanostructures in question is frequently dominated by the signal of its bulky companion, particularly with strong magnetic fields.

An ideal substrate or sample carrier should not introduce any signal during the measurements, but it always does during the integral magnetometry, so it should be of a simple form and easily accountable. This calls for materials of extreme purity whose (preferably) diamagnetic response should be perfectly linear in magnetic field *H* and independent of the temperature *T*. This is, however, a great technological challenge, and such materials either cannot be synthetized or their price exceeds budgets of typical research projects. This is probably why, ubiquitously, idealized magnetic properties of these substrates are assumed. Namely, their isotropic *T*-independent and linear in *H*- diamagnetic susceptibility is assumed and subtracted to obtain the searched response from the nanomaterial. Such an approach may even lead to qualitatively false conclusions. Even commercially available Si or GaAs wafers show disturbing nonlinear temperature- and field-dependent responses, particularly at cryogenic temperatures [4], yet systematic data are scarce, since it is hard to account for and report on effects where forms and magnitudes vary from sample to sample inconsistently.

Sapphire (Al_2_O_3_) belongs to the crystalline compounds whose chemical purity is still under development and is rather inferior to that of Si or GaAs, yet sapphire wafers are frequently chosen as the base for the deposition of nitride family structures and 2D materials for spintronics applications [5,6,7,8,9,10]. In the context of magnetism-oriented studies, sapphire substrates have been widely used to deposit dilute magnetic nitrides in an epitaxial manner, mainly GaN:Fe [11,12,13], GaN:Gd [14,15], and (Ga,Mn)N [16,17,18,19,20], to name the most prominent ones. The assumption of the ideal magnetic properties of sapphire is on one hand supported by results of theoretical calculations [21], and on the other by some experimental communication, e.g., [4,22]. However, there exists another body of experimental evidence pointing out that sapphire does exhibit pronounced *H-* and *T*-dependent contributions, e.g., [16,23,24,25]. Unattended, these strongly nonlinear in *T* and in *H* components will hinder accurate evaluations of the magnetic response of the structures under study, and may even lead to qualitatively false conclusions. Therefore, either great experimental care, backed by an adequate experimental routine [26,27], or a dedicated in situ compensation approach [25] is needed to properly assess and mitigate these detrimental contributions. It was found that this surplus magnetic response was predominantly caused by Cr present in the bulk of a material [23] or by a surface contamination by Fe [22]. More recent studies indicate that Er dopant has assumed the dominant role.

It has to be mentioned that the effects caused by contaminations due to handling, deposition processes and further processing can be even stronger than those pointed out above. In the first context, it has to be noted that sapphire is much harder than most of the tools used around the laboratory, so it acts as an abrasive material, eagerly accumulating small contaminations on the tool-crystal contact surfaces [22,28]. In the second context, the possibility of magnetic contaminations by residues of metallic glues or substrate backside metallization used to attach and/or thermalize the substrates in the growth chambers has to be brought to attention. Obviously, the magnitude of this spurious magnetic signal varies a great deal, but can easily exceed 10^−^^5^ emu at room temperature. These contaminations exhibit an overall sigmoidal or Langevin-like shape of their magnetization curves, which saturate swiftly at about 5 kOe [29,30,31]. The size of this effect makes it comparable to the magnitude of magnetic responses expected in magnetometry of nanomaterials, e.g., [3,32,33,34,35,36,37], and its ferromagnetic-like appearance could falsely constitute a basis to invoke the existence of ferromagnetism (FM) in the investigated nanomaterial.

Yet another important aspect of the nontrivial magnetic properties of the commonly met solid-state substrates comes to light when the magnetic anisotropy (MA) of the nanomaterials has to be investigated. Frequently, the form and the magnitude of MA provides indispensable information about the underlying processes in the investigated material. Equally importantly, a detailed knowledge of MA is required to validate some theoretical considerations [38,39]. This necessitates magnetic measurements in both in-plane orientation of *H* (the standard one), and (more experimentally cumbersome) the perpendicular one. This is in particular “a must-do” experiment in the case of aniferromagnetic materials, which, particularly in the form of very thin layers, are challenging to measure due to their rather weak magnetic response [40,41]. Finally, the evaluation of the magnetic properties has to be performed in the same experimental configuration in which the typical magneto-transport measurements are performed, i.e., having *H* oriented perpendicularly to the surface of the flat sample. As such, the presence of a significant MA in the substrate would considerably mar the outcome of the investigations if the relevant piece of the substrate had not been properly evaluated in the same experimental conditions.

Therefore, when orientation-dependent magnetic studies are performed, it is profitable to know whether the substrate material brings its own intrinsic and anisotropic contribution to the measurements. Having established early enough that the current substrate material exerts too strong magnetic anisotropy, one can either search for more appropriate substrates from other sources or modify the whole experimental approach to give a proper account of the contributions from the substrate. In this report, we put forward a simplified method of assessment of the magnitude of magnetic anisotropy of common substrates, considering sapphire as the sole example. It is argued that the method eliminates a large uncertainty brought about by most common artifacts related to the SQUID magnetometric systems and to the arrangement of the measurements.

This paper is divided in the following sections. We firstly introduce the material of our study and substantiate the needs for an alternative approach to precise volume magnetometry of nanostructures on bulky substrates. In Section 3, the results of our magnetic characterization of the sapphire specimens are given. They provide a solid justification for the method put forward by us. In Section 4, we detail the method and provide supporting results obtained from the modeling of a system of noninteracting ions exhibiting a sizable single-ion magnetic anisotropy. In Section 5, we enumerate a possible range of concentrations of magnetic ions that could be responsible for some of the leading paramagnetic contributions to the ideal diamagnetism of sapphire detected in our samples. The conclusions are given in Section 6.

## 2. Materials and Methods

The sapphire specimens investigated here originate from colorless single-side polished α-Al_2_O_3_ (hexagonal) epi-ready 2-inch wafers. They are all *c*-plane, i.e., the surface has the (0001) orientation, as required for epitaxial growth of various nitride structures that we have focused on in our research [11,13,18,20,42] for nearly two decades. The wafers were acquired from four different vendors. We label them “A” (6 samples), “B”, “C”, and “D”, since in this report we do not aim at any classification of the available material on the market. We concentrate only on the experimental validation of the method of a fast and reliable assessment of MA in nominally diamagnetic substrate crystals. The relatively broad origin of the investigated material helps us to generalize our conclusions.

The specimens are either cleft or professionally cut using a wheel saw from 2-inch wafers into approximately 5 × 5 mm^2^ pieces or into 1.3 × 5 mm^2^ strips to facilitate orientation-dependent studies, as detailed in Appendix A. All the pieces are etched in an ultrasonic bath of HCl for about 15 min to remove surface and postprocessing contamination. All magnetic measurements are performed between 2 and 350 K and up to 50 kOe in a quantum design (QD) MPMS SQUID magnetometer equipped with a low field option (allowing the magnet reset feature). Sufficiently high signal-to-noise ratio of the results is facilitated by the use of the reciprocating sample option (RSO). For the SQUID measurements, the specimens are glued using strongly diluted GE varnish [43] to sample holders made of 2 × 0.7 mm^2^ across and about 19 cm long Si sticks professionally cut from industrial 8-inch wafers [26]. The use of Si sticks, employed routinely by the authors for their most sensitive magnetometry studies [25,33,44], completely eliminates position-dependent magnetic signals commonly observed when plastic straws are used to fix the sample in SQUIDs [45,46]. In the authors’ view, the use of plastic straws, in particular “straight from the box”, i.e., without any selection and testing prior to the measurements, is highly inadvisable and should be avoided in precise, high-sensitivity magnetometry. In the same context, the usage of Si sticks, as well as quartz paddle sample holders in the QD VSM-SQUID, facilitates perfectly reproducible sample positioning, which is a prerequisite condition for the conventional approach to studies of very weak magnetic anisotropies.

Since the purpose of this report is rather practical, we present our results normalized to a standard, approximately 5 × 5 × 0.3 mm^3^, sapphire piece weighing 30 mg and express them in the experimental units of the magnetic moment *m* (emu). This will allow an easy and direct comparison of the results presented here with unprocessed results in other studies. Such a 30 mg sapphire specimen exerts about −10^−4^ emu at 10 kOe.

Undoubtedly, the main experimental challenge in volume magnetometry of nanomaterials is caused by the fact that the signal of the bulky substrates is rather strong and increases linearly with *H* towards |*m*| = 10^−3^ emu at 70 kOe, whereas that of the nanostructures rarely exceeds 10^−5^ emu, and frequently is much weaker. Therefore, all magnetic measurements have to be carried out by strictly observing the experimental code [26] elaborated to eliminate artifacts and to evade limitations associated with integral SQUID magnetometry [27]. Nevertheless, a simple subtraction of the diamagnetic component originating from the sapphire substrate under an assumption that it is linear in *H* only exposes the resulting data to various artifacts related to the SQUID system and to arrangements of the measurements [24,47]. Other approaches are needed. One of the best methods to mitigate these artifacts is a direct in situ compensation of the signal of the substrate [25,48] or of the carrier [49]. Another could be the physical separation of the material under study from the troublesome substrates [31,35,50,51,52]. However, the latter approach necessitates affixing the separated material onto another, substantially cleaner material. Anyway, a sizable disparity between the signal of interest and that of the new carrier may still occur. Therefore, one has to resort to an independent assessment of the magnetic anisotropy of this new supporting material. In this paper, we put forward a method that simplifies this task. It allows a much faster and more accurate, though relative, assessment and provides an economical way of ranking of the available material(s) intended to be used as substrates, supports, or carriers for studying magnetic anisotropy in nanostructures.

## 3. Results and Discussion

**Figure 1:**
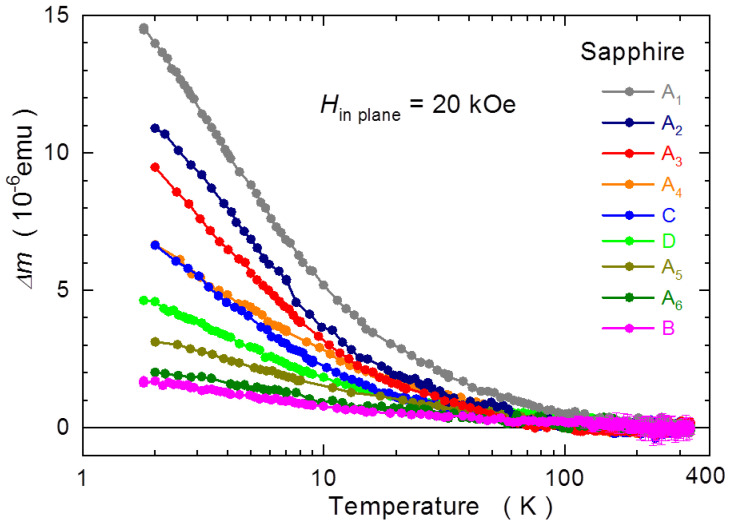
Temperature variation of the magnetic moment *m* in various sapphire samples originating from four different vendors, A to D, as indicated in the legend. The results for all the samples are rescaled to correspond to a 30 mg sample and the temperature induced changes Δ*m* are calculated with respect to *m*(300 K). Magnetic field *H* of 20 kOe is applied in the plane of the samples, i.e., *H* is perpendicular to the *c* axis of these *c*-plane sapphire specimens. For clarity, the error bars are indicated only for sample B.

Figure 1 shows the range of variations Δ*m*(*T*) = *m*(*T*) − *m*(300 K) from the expected temperature-independent behavior established for the range of sapphire samples considered here. All these measurements are performed at *H* = 20 kOe applied in the plane of the samples (that is, for *H* being perpendicular to the wurtzite *c* axis of *c*-plane sapphire). The reference value, *m*(20 kOe, 300 K), of a 5 × 5 × 0.3 mm^3^ (~30 mg) piece of sapphire is about −2.2 × 10^−4^ emu. More exactly, the mass susceptibility *χ* of the samples studied here is *χ*_Sapphire_(300 K) = −3.7(1) × 10^−7^ emu/gOe. This value has been obtained taking the sample-to-SQUID coupling factor *γ* = 0.983 appropriate for a 5 × 5 mm^2^ sample investigated in the in-plane configuration [26]. We need to modify the results reported by magnetometers by a size- and orientation-dependent coefficient *γ*, which rescales the response of the SQUID software, calculated in the point object approximation, to the real response of a physical objects of given dimensions [26,53]. The logarithmic scale of temperature is used only for convenience. We want to expose the low *T* region, where most of the changes take place. Indeed, down to 100 K, hardly any changes are seen on any Δ*m*(*T*) curves. Curie-type paramagnetic-like deviations develop around 100 K, and at 2 K this contribution can be as strong as 2 × 10^−^^5^ emu. This is actually as much as about 10% of the diamagnetic response of sapphire.

It has to be pointed out that on the grounds of theoretical considerations, no temperature dependence of *χ* in sapphire has been expected [21,54]. This stems from the huge energy gap of sapphire, *E*_g_ ≅ 9.9 eV, for which the only *T*-variable contribution to *χ*, the van Vleck-type paramagnetism, *χ*_vV_, determined by the *T*-dependence of the bandgap, is practically negligible, since *χ*_vV_ ∝ 1/*E*_g_. Historically, both the *T*-independent properties [4,46] and *T*- and *H*-dependent intrinsic magnetism in similar sapphire samples [16,23,24,25] were noted. The results collected in Figure 1 clearly contradict the former claims. In neither case is Δ*m*(*T*) independent of *T*. In fact, all Δ*m*(*T*) measured by us indicate the existence of a variable in size Curie-like paramagnetic contribution. The overall picture emerges that there is not any “universal sapphire” material. The magnetic response of sapphire substrates available on the market changes from sample to sample and the differences among them are substantial. This is particularly clearly seen within the range of “A” samples, by far the most numerous species in our study. These samples cover (nearly) the full range of Δ*m*(*T*) observed here. The material from other sources shows rather smaller magnitudes of Δ*m*(*T*), yet still with a sizable distribution. This important finding indicates that commercially available sapphire is not magnetically clean and that researchers must take for granted the existence of a certain level of paramagnetic-like contaminants. Lastly, we underline here the most worrying fact of a rather large magnitude of these paramagnetic deviations. If left unaccounted, such a behavior of a substrate is sure to mislead any low-temperature nanomagnetic studies.

**Figure 2:**
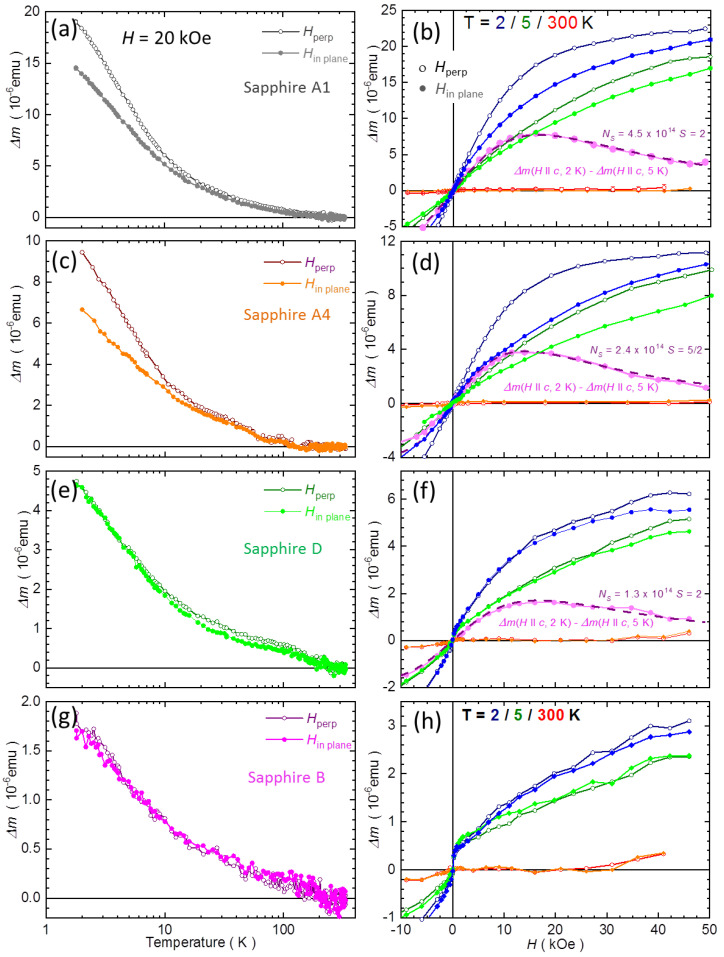
Results of the magnetic field *H* orientation-dependent studies of selected four sapphire specimens in temperature *T* domain (left panels) and in *H* domain (right panels). Each pair (**a**,**b**), (**c**,**d**), (**e**,**f**), and (**g**,**h**) presents results for one of these samples. Results in *T* domain are obtained at *H* = 20 kOe. Here, the results are normalized to correspond to a 5 × 5 × 0.3 mm^3^ (~30 mg) sapphire sample, and Δ*m*(*T*) = *m*(*T*) − *m*(300 K). The results in *H* domain are plotted after the application of a linear correction *α·H*, where *α* is the slope, of *m*(*H*, 300 K); Δ*m*(*H*) = *m*(*H*) − *α·H*. Open symbols stand for perpendicular orientation *H*, the closed ones represent *H* applied in the plane of these *c*-plane sapphire samples. The magenta bullets in *H*-panels (**b**,**d**,**f**) show the difference between Δ*m*(*H*_perp_) measured at *T* = 2 and 5 K to evaluate an approximate number *N_S_* of spins *S* in the sample. The dashed purple lines represent the fit of difference between Brillouin functions for given *S* at 2 and 5 K. The possible magnitudes of *N_S_* and *S* yielding the best fit are given in the panels.

Actually, the magnitudes of the changes of Δ*m*(*T*) reported in Figure 1 tie to a large degree with anisotropic properties of these samples. The temperature-dependent data for both the in-plane orientation of *H*, *H*_in plane_, and for *H* applied perpendicularly to plane, *H*_perp_, are exemplified in the left panels of Figure 2. Here, Δ*m* is obtained similarly to the results presented in Figure 1, i.e., the magnetometry data are normalized to 30 mg and additionally rescaled to yield Δ*m*(*T*) = 0 at room temperature for both orientations of *H*. The following general pattern emerges. A clear magnetic anisotropy is seen in these samples, which exhibits the largest values of Δ*m*(*T*), say, above about 5 × 10^−^^6^ emu at the lowest temperatures. This is above about 2% of the pure diamagnetic response of sapphire. When magnitudes of Δ*m*(*T*) are below this limit, no dependence on the orientation of *H* is registered within experimental accuracy, as exemplified in panels (e) and (g) of Figure 2. It remains an interesting question if this general picture holds also for other sapphire substrates and whether, or not, a similar threshold value could be identified. Another general result of our scrutiny is that in all cases when we see MA, the positive correction to *m*, Δ*m*(*T*, *H*), is stronger for *H*_perp_ than for *H*_in plane_. Or Δ*m*(*T*, *H* || *c*) > Δ*m*(*T*, *H* ⊥ *c*), when one refers to the crystallographic orientations. We dub this case the “negative MA,” since the net paramagnetic contribution to *m* in this by far more frequently exercised orientation in magnetometry, i.e., with *H* applied in the plane of the substrate, is smaller than that when *H* is applied perpendicularly to the face of substrate.

Magnetic measurements performed in the magnetic field domain do confirm the conclusions of the *T*-dependent results, as shown in the right panels of Figure 2. Here, we consider only the nonlinear parts of *m*(*H*), Δ*m*(*H*) = *m*(*H*) − *α·H*, where *α* is the slope of *m*(*H*, 300 K). Magnitudes of *α* are established separately for each sample and each orientation of *H*. Indeed, each measurement run yields its own value of *α*, In practice, *α* is not directly proportional to *χ*_sapphire_(300 K). This is because the results of the measurements (the bare numbers reported by the magnetometer) depend also on the magnitude of the orientation-dependent coupling factor *γ.* Equally importantly, *α* also contains all other factors much harder to account for, which additionally influence the absolute values of *m* reported by the magnetometer. The first ones are related to an imperfect sample positioning. These include both the rotational and the radial misalignments of the specimen in the SQUID sample chamber with respect to the centerline of the magnetometer. These sources of errors are poorly described in QD technical notes for MPMS systems. One should refer to SQUID VSM technical notes for more details [55,56]. The other frequently occurring errors are brought about by the deviations from the ideal performance of the RSO sample transport mechanism. Any of these factors alone can influence the magnitude of the response of the magnetometer by up to 2%. In this very sense, *α* parameters are not sample-specific quantities: they are specific to each experimental run, as they reflect the current experimental geometry, specimen (mis)alignments, and the performance of the mechanical parts of the magnetometer. Therefore, a different value of *α* is frequently obtained when the same sample is remeasured in nominally the same arrangements.

The results collected in panels (b), (d), (f), and (h) confirm that extra magnetic response of all the sapphire samples studied by us is a paramagnetic-like. However, we can identify two leading patterns. Samples with large values of Δ*m*(*H*) exhibit a smooth Brillouin-like response, yet with a pronounced MA. Similarly to the corresponding *T*-dependent results, the *H*_perp_ orientation is the easy one. The results shown in the lowest panel (h) exhibit a strong upturn of *m* around *H* = 0, which is followed by a pronounced kink. They do not show any tendency for saturation up to 50 kOe, and MA, if any, is very weak. The different origin of this behavior is also indicated by the fact that the magnitudes of Δ*m*(*H*) in Figure 2h are the smallest. It can be noted that both Δ*m*(*H*) in panel (f) constitute a kind of border case, as they contain both the Brillouin-like and the non-Brillouin-like types of Δ*m*(*H*), with clear dominance of the former. Actually, the Δ*m*(*H*) reported in panels (f) and (h) are very much alike those reported in Figure 1 of ref. [16], although the source of these wafers is different.

The case presented in panel (h) illustrates the fact that Δ*m*(*H*) of the epi-ready sapphire substrates may exhibit a deceivingly FM-like character. However, actually no other typical FM features like magnetic hystereses or nonzero remnant moments have been observed in this and all other investigated specimens. This is a substantially different outcome than that of [22,29], where a sizable concentration of Fe or Ti (mostly on the surfaces) was identified to be responsible for strong hystereses of Δ*m*(*H*). This fact underpins the importance of proper sample cleaning prior to the measurements and highlights the use of nonferrous tools to handle the wafers and the samples.

Similarly to the results of the *T*-dependent studies, MA is clearly seen in the top two right panels of Figure 2, and practically no anisotropy is seen in the two lower ones. This obvious correspondence between Δ*m*(*T*) and Δ*m*(*H*) forms the basis of our simplified method of the assessment of the magnetic anisotropy in common semiconductor substrates. Instead of typical measurements in the magnetic field domain, which are much longer and more demanding in terms of required precision, reproducibility, and signal-to-noise ratio, we suggest performing measurements only in the temperature domain, which are generally simpler, less noisy (magnetic field is stable within hours-long *T*-sweeps), and much faster to acquire. The latter means they are also more economical in terms of a higher throughput and lower helium consumption.

Therefore, we postulate that a sufficiently accurate magnetic evaluation of a family of similar semiconductor substrates, including the assessment of the (relative) strength of their magnetic anisotropy can be done on account of temperature-dependent measurements. As modeled below, just two temperature sweeps in a relevant temperature range performed in the two required orientations of *H* are sufficient to perform this task. The existence of MA, or its absence, will be clearly seen in the combined plot of Δ*m*(*T*, *H*_⊥_) and Δ*m*(*T*, *H*_||_). If MA is present, the enumeration of the area between these two Δ*m*(*T*) curves will quantify its strength and so will allow the classification or ranking among the specimens (substrates) in the study.

## 4. Description of the Method

The key factor underlying the method proposed here is that MA of weakly contaminated solid-state substrates is negligible at elevated temperatures and develops only at cryogenic ones, discarding cases of extrinsic contaminations, e.g., [29,30,31]. Under our assumption, the high-*T* magnitudes of *m*(*T*) should be the same for both *H*_⊥_ and *H*_||_, providing us with a very useful normalization feature. This normalization is the key point here, since it allows mitigation of the substantial inaccuracies resulting from different experimental arrangements specific to separate measurements in different orientations of *H* [26,53,55,56]. In particular, after the normalization, each pair of the *T*-dependent measurements will not be affected by the recent experimental history of the magnetometer. As a result, these two measurements in question do not have to be performed one after another, though this is an advisable option. The normalization also makes the analysis much easier and increases the confidence level of the results.

In the classical approach, the magnitude of MA can be obtained from the integration of the difference between the easy and hard axis magnetization curves. Here, we suggest enumerating the area *A_T_* between Δ*m*(*T*) established for two relevant orientations of *H*. *A_T_* does not carry its usual meaning of magnetic anisotropy energy density, but its magnitude can be used to very good ends as a numerical quantifier to rank a range of specimens among themselves, as is demonstrated further below.

To substantiate our method, we model theoretically an equivalent material system: wurtzite GaN:Mn. Importantly here, Mn substituting Ga in GaN assumes 3+ oxidation state, which due to *L* = 2 and *S* = 2 of its 4 electrons on the *d*-shell exhibits a strong single-ion magnetic anisotropy with respect to its wurtzite *c* axis [16,57]. GaN:Mn, as sapphire, is also highly insulating. In fact, it has been strongly researched recently as an ideal insulating substrate for homoepitaxy of planar high-power nitride devices [58]. In the paramagnetic concentration range, say, below 10^19^ cm^−3^, *T*-, *H*-, and orientation-dependent magnetic properties of GaN:Mn are adequately described within the crystal field model of *d*^4^ ion in the wurtzite host [16,39,59,60]. We follow this approach to compute exemplary *m*(*H*) and *m*(*T*) curves for various strengths of the single-ion MA. The latter depends predominantly on the magnitude of the trigonal distortion (along the *c*-axis) and in fact the strength of MA in GaN:Mn has been documented to change by direct magnetic measurements of piezoelectrically modulated structures [39]. The strength of MA and the shape of the relevant *m*(*H*) curves also depend strongly on the occupancy of the *d* level [38], what additionally makes the results of the modeling presented below relevant to our studies.

**Figure 3:**
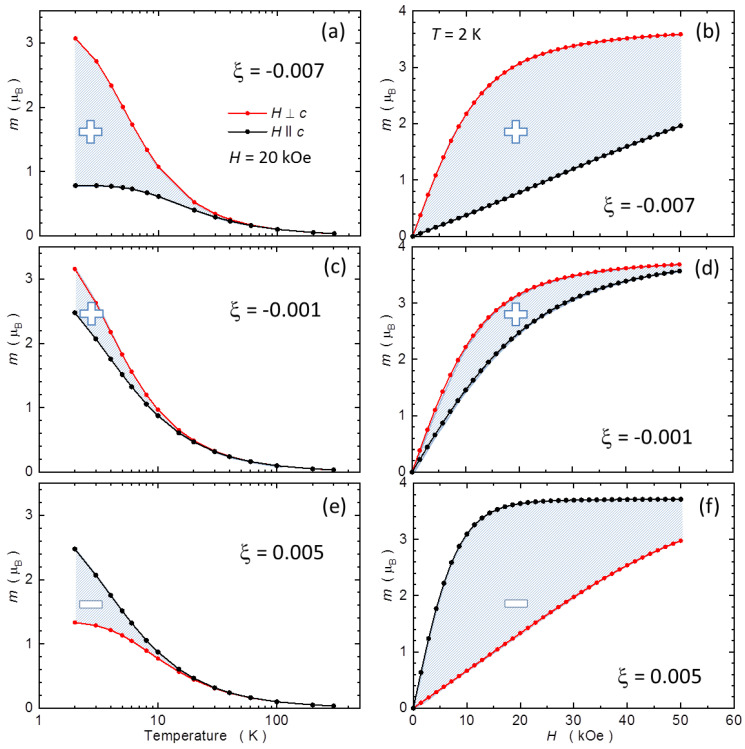
Single-ion magnetic anisotropy for Mn^3+^ ion in GaN calculated as a function of temperature *T* at *H* = 20 kOe [left panels (**a**,**c**,**e**)], and as a function of magnetic field *H* at *T* = 2 K [right panels (**b**,**d**,**f**)] for a model system of wurtzite GaN:Mn. The red symbols represent the *H* ⊥ *c* orientation, the black ones the *H* || *c* one, where *c* is the wurtzite hexagonal axis. The magnetic moment *m* is expressed in Bohr magnetons. We define the positive magnetic anisotropy when *m*(*H* ⊥ *c*) > *m*(*H* || *c*). The corresponding signs are indicated in each panel. The key model parameter here is the magnitude of *ξ* = *c/a* − (8/3)^1/2^, which quantifies the relative magnitude of the trigonal deformation from the ideal wurtzite structure [39].

**Figure 4:**
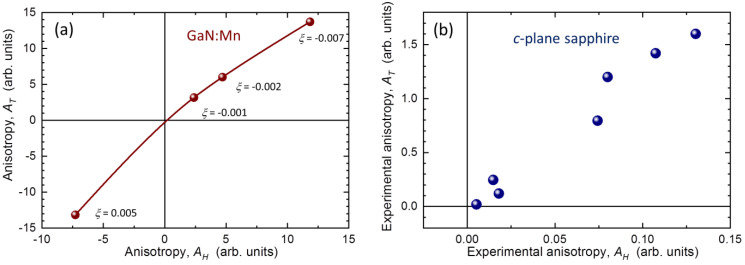
(**a**) Magnitudes *A_H_* and *A_T_* of the hatched areas between the branches of *m*(*T*) and *m*(*H*) presented in Figure 3. Their signs represent the orientation of the magnetic anisotropy according to the labels in Figure 3a. The solid line is a guide for the eye. (**b**) The same as in (**a**), but calculated from experimental data for some of the sapphire samples tested in this study, including those presented in Figure 2. The sign conversion adopted here indicating the positive magnitudes of *A_H_* and *A_T_* in panel (**b**) means that the easy axis orientation of the experimental magnetic anisotropy in *c*-plane sapphire samples is perpendicular to the sample plane (*H* **||** *c*).

The results of the modeling of MA in GaN:Mn are collected in Figure 3 and summarized in Figure 4a. We have checked that qualitatively the results do not depend on the choice of the strength of *H* from the range of fields available in commercial magnetometers. Therefore, for brevity we present only the results obtained for *H* = 20 kOe, the same field as used to establish experimental Δ*m*(*T*) in sapphire. The strength of the trigonal distortion can be quantified by a parameter *ξ* = *c*/*a* − (8/3)^1/2^, i.e., by the relative magnitude of the trigonal deformation from the ideal wurtzite structure [39]. The pairs of panels (a,b), (c,d), and (e,f) in Figure 3 exemplify cases of *ξ* = −0.007 (corresponding to free-standing GaN:Mn, i.e., a slightly compressed wurtzite crystal along its c-axis), *ξ* = −0.001 (a nearly ideal wurtzite), and *ξ* = 0.005, a uniaxially elongated GaN:Mn, respectively. The reversed strengths of the two branches of *m*(*H*) in panel (f) with respect to the branches in panels (b) and (d) indicates the change of sign of the magnetic anisotropy for *ξ* > 0. Most importantly here, the same reversed strength is seen in panel (e), so the change of the sign of MA is equally clearly reflected in the temperature domain. We now enumerate the differences between each pair of branches within the experimental ranges of *H* and *T* by calculating the corresponding areas, *A_H_* and *A_T_*, respectively (indicated in Figure 3). The integration in *H* domain is limited at *H_max_* = 50 kOe, the lower limits of *H* available in MPMS SQUID magnetometers. We confirm that the results are qualitatively the same if *H*_max_ = 70 kOe is adopted. The resulting dependence of *A_T_* on *A_H_* is plotted in Figure 4a. The points collected there show that indeed a quantification of the strength of MA on the account of *A_T_* yields qualitatively the same results as using measurements in the field domain. Let us point out once again that the analysis of results in temperature domain does not provide absolute values of MA, yet it correctly yields its sign and is more experimentally efficient. In practice, the choice of *H* should take into account both the expected magnitude of Δ*m*(*T*) and the noise level of the system. In the case reported here, anything between 10 and 40 kOe is satisfactory, since MA is sufficiently developed and the noise from the superconducting magnet remains within acceptable limits. The quality of the experimental evaluation deteriorates on moving to stronger fields: the magnitude of MA drops and the SQUID magnetometer yields more noisy readings.

To conclude this part, we plot in Figure 4b the magnitudes of *A_T_* versus *A_H_* for some of the investigated sapphire substrates. The points form a very similar pattern to that seen in the top-right quarter on panel (a). Firstly, it means that indeed the integration in the *T*-domain can be used for quantification of MA in such materials like sapphire. Secondly, the points in panel (b) tend to bunch around (0, 0) and at higher values of *A_T_* and *A_H_*. This indicates that in the whole range of the investigated *c*-plane sapphire substrates, either there is practically no MA or a relatively strong MA develops at low temperatures. In this case, the paramagnetic change of *m* is greater for the perpendicular orientation of *H* than for the in-plane configuration. It remains to be seen whether other sapphire crystals can exert also a reversed MA, or the pattern observed here is a universal one for these commercial substrates.

## 5. Quantification of the Strong Paramagnetic Component

Finally, we attempt to shed some light on the origin of the strong Brillouin-like form of experimental Δ*m*(*H*), shown in panels (b), (d), and (f) of Figure 2. The character of Δ*m*(*H*) reported there resembles greatly that of a single-ion magnetic anisotropy, as presented in our numerical modeling, panels (b), (d) and (f) of Figure 3. However, nothing is known about the oxidation state and the exact coordination of these ions, so we cannot rely on the precision of the crystal field model. Therefore, we resort to the classical Brillouin function *B_S_*(*H*, *T*):

$$B_{S}\left(x\right)=\frac{2S+1}{2S}coth\left(\frac{2S+1}{2S}x\right)-\frac{1}{2S}coth\left(\frac{1}{2S}x\right),$$

with *H* and *T* tied in *x* by:

$$x=S\frac{g\mu_{B}H}{k_{B}T},$$

to model the magnetic response of these ions. Here, *g* = 2 is the Land’e factor, *μ*_B_ is the Bohr magneton, and *k*_B_ is the Boltzmann constant. However, we know that there exists another admixture to experimental Δ*m*(*H*), which dominates in Figure 3h. Since we cannot account numerically for this contribution, we employ a technical trick outlined below. It allows a separation of the PM contribution from the other magnetic sources, usually exhibiting a FM-like form of their *m*(*H*), like a response of FM nanocrystals embedded in the paramagnetic matrix [13,49,61,62], or a form of the Langevin function exerted by superparamagnetic-like phase-separated mesoscopic volumes [31].

In the first step of our approach, we enumerate the experimental difference between the magnetic isotherms measured at 2 and 5 K, Δ*m*(*H*, Δ*T*) = Δ*m*(*H*, 2 K) − Δ*m*(*H*, 5 K). The results are marked in the corresponding panels by magenta bullets. The main point here is that Δ*m*(*H*, Δ*T*) is largely devoid of the non-PM components, since the *T*-induced changes of *m*(*H*) at low temperatures are the strongest for paramagnets. The other components largely cancel each other out. In the last step, we fit to these Δ*m*(*H*, Δ*T*) the difference of two Brillouin functions taken at the same temperatures as above. Namely, we fit $N_{S}g\mu_{B}S\left[B_{S}\left(H,2\;K\right)-B_{S}\left(H,5\;K\right)\right]$, where *N_S_* is the number of ions with spin *S* = *n* × 1/2. Both *N_S_* and *n* are the only adjustable parameters. The results of the fits are indicated by dashed lines, indicating that a very reasonable fit can be obtained in all three cases, i.e., when Δ*m*(*H*) is dominated by the smooth PM contribution. The magnitudes of *N_S_* and *S* are given in the corresponding panels. As said, these values cannot be treated as exact numbers, but we can safely conclude that the spin state of these ions is close to *S* = 2 (for *g* = 2) and that the number of these ions increases from 1 × 10^14^ to 4 × 10^14^ (their concentration changes from 1.3 to 6 × 10^16^ cm*^−^*^3^, respectively) going from sample D to A1, as indicated by the increasing magnitudes of Δ*m*(*H*) along these three samples. We did not apply this procedure to the data in Figure 2h, since these Δ*m*(*H*) values cannot be approximated by classical Brillouin function. It is beyond the scope of this report to pursue the origin of this rather intriguing Δ*m*(*H*).

## 6. Conclusions

A simplified method of the assessment of the magnetic anisotropy in solid-state substrates has been presented. Its applicability has been validated by magnetic investigation of common sapphire epi-ready wafers available on the market. It has been experimentally evidenced that in order to acquire a qualitatively correct information on the magnetic anisotropy of the material, instead of technically more cumbersome measurements in the magnetic field domain, one can resort only to temperature-dependent studies. The presented experimental data and the theoretical modeling substantiate the fact that qualitatively correct information about of the strength and the sign of the magnetic anisotropy can be obtained by integration of the difference in results of two temperature-dependent measurements performed for two relevant orientations of the magnetic field. This allows for faster, more reliable and economical efficient classification or selection of the material in possession, or to select the vendor with the most suitable substrates for the planned study. Very importantly, the method detailed here is practically immune to all the already-recognized factors that mar precision volume magnetometry performed in commercial SQUID magnetometers. This fact additionally saves experimental time and confusion and allows obtaining reliable outcomes, even by a novice in magnetometry.

Although the method has been tested and validated for *c*-plane sapphire wafers, the authors are confident that it can be applied to other types of sapphire substrates and crystalline solids, such as Si, GaAs, etc., for which the body of experimental evidence will point out to a convenient temperature range in which the normalization of the results obtained in different orientations of the magnetic field can be performed.

Concerning sapphire, our detailed study indicates that most likely all the *c*-plane sapphire substrates that have been available on the market do not exhibit purely diamagnetic responses. They exhibit a net paramagnetic contribution of various strengths. We find that this contribution can be as strong as about 2% of the pure diamagnetic response of sapphire, rigorously evaluated here to be *χ*_Sapphire_(300 K) = −3.7(1) × 10^−7^ emu/gOe. In absolute numbers, this paramagnetism can bring as much as 2 × 10^−5^ emu at 20 kOe, a number that easily exceeds the magnitudes of the magnetic responses seen in nanoscale structures, including 2D materials. This is the range of numbers that one should really reckon with, and it indicates that the adequate magnetic testing of the substrate material should be mandatory in magnetometric studies of miniscule nanostructures deposited on sapphire substrates. It has been evaluated that this paramagnetic component may be brought about by some 10^16^ cm^−3^ transition metal ions. More importantly, our study indicates that sapphire crystals exhibiting net paramagnetic responses exceeding about 2% of their diamagnetism exert a magnetic anisotropy with the easy axis oriented along the *c* wurtzite axis of sapphire, i.e., perpendicular to the plane of the *c*-plane substrates. Additionally, yet another paramagnetic-like contribution in sapphire has been identified, which is seen only when the concentration of the transition metal ions is very small. It exhibits a much more ferromagnetic-like shape of its magnetization curves, but it is devoid of magnetic anisotropy. The origin of these spurious contributions to the diamagnetism of sapphire remains to be unraveled.

Our findings form an important message that this nonstandard magnetism of commonly available substrates can significantly influence the results of precise magnetometry of nanoscale materials and that its existence must be taken for granted by both industry and academia.

## Figures and Tables

**Figure A1:**
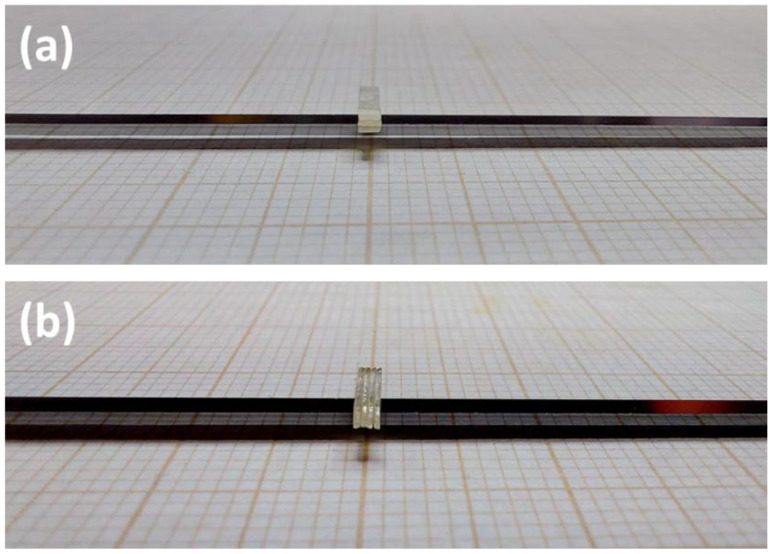
An example of the modification of a 5 × 5 mm^2^ piece of 0.3 mm-thick sapphire sample into a symmetrical assembly of approximately 1.3 × 1.3 × 5 mm^3^. The sample is mounted onto a long Si stick serving as the final part of the QD MPMS typical sample holder for RSO measurements. (**a**) The sample is mounted for the in-plane measurements (*H* is parallel to the surface of the original piece), and (**b**) the same sample is mounted for the perpendicular orientation.

## Data Availability

The data presented in this study are available upon reasonable request from the corresponding author.

## Appendix A

In order to largely reduce the detrimental influence of the sample geometry on the results of the measurements of the magnetic anisotropy, it is advisable to prepare the sample in a shape that has the same *γ* factor in both relevant orientations in the magnetometer. Again, the problem is in the large magnitude of the signal of the substrate. It can be as large as about −10^−3^ emu in 70 kOe, so a change in the orientation of a 5 × 5 mm^2^ specimen from the in-plane into the perpendicular orientation causes changes in the coupling to the SQUID pickup coils from 0.983 to 1.034 [26]. Accordingly, even for an isotropic material, the signal reported by a MPMS SQUID magnetometer will change by 5%, which is about 5 × 10^−5^ emu in this example.

To minimize this massively detrimental effect, we are preparing our samples by cutting the material into narrow strips of approximately 1.3 × 5 mm^2^. Stacking four of them on top of one another forms a cuboid with cross-section approximately 1.3 × 1.3 mm^2^ [39]. Now, any rotation by 90° along the long side of the cuboid facilitates a change in orientation between the in-plane and the perpendicular one with marginally weak changes in the *γ* coupling factor, thus largely preserving the correspondence of the data collected in both orientations. The experimental realization is exemplified in Figure A1.
